# Short-term neurological and functional outcome of surgical intervention in spinal cord injuries: a single center prospective observational study

**DOI:** 10.11604/pamj.2023.45.138.37180

**Published:** 2023-07-21

**Authors:** Tuhin Purkayastha, Anupam Debnath, Sankar Debroy, Sachlang Debbarma

**Affiliations:** 1 Agartala Government Medical College, Tripura, India

**Keywords:** Spinal cord, neurological, recovery, rehabilitation

## Abstract

**Introduction:**

the management of an acute spinal cord injury remains controversial. The patient of acute spinal cord injury undergoes several phases of care beginning with the initial trauma management, surgical intervention, and perioperative medical management. The aim of this study was to evaluate the neurological and functional outcome of operative management of traumatic spinal cord injury patients admitted to a tertiary care centre in Northeast India.

**Methods:**

thirty patients with spinal cord injury admitted to a tertiary care centre from December 2019 to November 2021, and treated with instrumented stabilisation for spinal cord injury were evaluated until 6 months postoperatively. Patients were evaluated with validated neurological (American Spinal Injury Association scale) and functional outcome measures (Barthel index). Demographic details, mode of injury, morphology, patterns of fractures, neurological level, and management methods in the hospital were recorded and analysed using the Statistical Package for the Social Science (SPSS) version 27.0.

**Results:**

thoracolumbar spinal cord was more commonly injured with 16 (53.3%) patients compared to cervical spinal cord injury patients at 14 (46.7%). Eight patients had complete recovery, 7 patients had incomplete recovery and 15 patients had no recovery. At 6 months post-injury, 18 (60%) patients had favourable functional outcome. American Spinal Injury Association (ASIA) grade at admission was found to be significantly associated with the functional outcome.

**Conclusion:**

after surgery half of the patients had an improvement in their neurology, and functional outcome was favorable which suggests that surgery still holds the key to a better functional and rehabilitation outcome.

## Introduction

The spinal cord is a long cylindrical extension of the central nervous system situated within the vertebral column. The symptoms of spinal cord lesions depend on the extent of the injury. Cervical spinal cord injury commonly causes sensory and motor loss (paralysis) in the arms, body, and legs, a condition called tetraplegia or quadriplegia while thoracic spinal cord injury commonly causes sensory and/or motor loss in the trunk and legs, a condition called paraplegia [1]. Epidemiologic data on spinal cord injuries in India are sparse. The low socio-economic status and younger age group had a major financial, social, and psychological impact as the majority of the patients were the primary earning members of the family [2]. Despite progress in management, spinal trauma is still a source of significant morbidity with a pronounced decrease in quality of life [3]. The management of an acute spinal cord injury remains controversial. The patient of acute spinal cord injury undergoes several phases of care beginning with the initial trauma management, surgical intervention, and perioperative medical management [4].

Spinal cord injury encompasses a cascade of events that begins with a primary mechanical injury followed by a secondary pathological response, the main determinants of severity being the extent of initial destruction and duration of spinal cord compression [5]. An important aspect is the early optimization of the patient for surgical decompression and stabilization since the timing of surgical intervention is critical to maximize potential outcomes [6]. Medical and pharmacologic management of spinal cord injury remains relatively controversial. Recent research has focused on the postinjury inflammatory cascade, with clinical trials underway for a range of neuroprotective agents [7]. The mainstay of treatment of acute spinal cord injury remains early surgical intervention. In animal models, decompression of the spinal cord after durations of persisting compression of up to 4 hours simulating those encountered in clinical practice suggested neurological recovery [8]. Decompressive study is indicated within 24 hours of acute traumatic spinal cord injury [9]. Surgical stabilization in the case of cervical spinal cord compression or instability is often the treatment of choice to grant a chance of neurological recovery, early mobilization, and a faster return to usual daily activities compared to the conservative treatment [10]. The treatment goals for thoracic or lumbar fracture dislocations are to achieve reduction, immediate stabilization with spine fusion, neural element decompression, and early rehabilitation [11].

Since there is no curative treatment yet for spinal cord injuries, prevention is paramount, and investigation of the demographic profile is the first step in planning for preventive strategies. To assess the demographic profile of patients with traumatic spinal cord injury attending a tertiary care center in Northeast India and to gain a deeper understanding of the results and problems associated with operative intervention this study was undertaken.

## Methods

**Design and setting:** a prospective observational study was conducted over a 2-year study period after getting approval from the Institutional Ethical Committee on 30 adult patients of traumatic spinal cord injury who underwent surgical intervention at Agartala Government Medical College and GB Pant Hospital in Agartala, Tripura, India from December 2019 to November 2021. Concomitant head injuries, penetrating injuries to the spine, pre-injury major neurologic deficit, and polytrauma patients were excluded from the study. The sampling method followed was convenience sampling.

**Study participants' characteristics/procedure:** after presentation to our centre patients were resuscitated following standard Advanced Trauma Life Support (ATLS) protocol and radiological examinations (X-ray, computed tomography (CT) scan, and magnetic resonance imaging (MRI) with screening of whole spine) were obtained after achieving haemodynamic stability according to the needs of the patients. At presentation neurological examination was performed and recorded as per standards established by the International Standards for Neurological Classification of Spinal Cord Injury (ISNCSCI) or American Spinal Injury Association (ASIA) classification, a universal classification tool for spinal cord injuries based on a standardized sensory and motor assessment. Data were collected from the filled proformas, medical and operative records of the patients.

**Operative procedure:** in all the patients who gave consent for the operation, routine investigations for anaesthetic evaluation of the patient were done as per the hospital protocol. The proforma was maintained for each patient to record the particulars and to keep a record of the variables concerned. In our study, the decision on surgical timing was dependent on the time required to obtain diagnostic investigations. The specifics of the surgical intervention, such as the direction of approach (anterior vs. posterior) and a number of levels decompressed, were also decided based on the judgment of the attending spinal surgeon. In all cases, decompression was accompanied by an instrumented fusion procedure.

Methylprednisolone was used at the discretion of the treating team according to the recommendations of the National Acute Spinal Cord Injury Study (NASCIS)-2 study [12]. For cervical spine injuries operative stabilization was performed by both anterior and posterior approaches. For thoracolumbar spinal injuries, stabilisation was performed by posterior approach. All patients underwent a post-operative rehabilitation regimen, tailored to individual and injury-specific factors. Documentation of postoperative complications was also performed.

**Anterior approach to the cervical spine:** the patient was placed in 20 degrees of reverse trendelenburg positioning, to allow for venous drainage with a small roll placed between the scapulae. Gentle caudal traction to the shoulders was applied using tape. A vertical incision was made parallel to the sternocleidomastoid (SCM) and approximately 1 cm medial to the medial border of the muscle. Sharp dissection was made down to the platysma muscle. The platysma was divided horizontally. Deep to the platysma, the deep cervical deep cervical fascia was incised with the platysma and then elevated away from the SCM and strap muscles. The pretracheal fascia was entered by blunt dissection vertically along the medial border of the sternocleidomastoid. The appropriate level was verified by using a marker and a lateral radiograph. Staying directly on the bone and disk annulus, the longus colli muscle was elevated away from both sides. Laterally the dissection was carried out till visualization of the uncovertebral joints. After the longus colli muscle was elevated, self-retaining retractors were placed and diskectomy was performed. Although this approach was generally associated with minimal blood loss, a drain was placed to ensure that a hematoma does not develop over the first night. The platysma muscle and skin were closed separately using interrupted sutures. The drain was removed on postoperative day 1.

**Posterior approach to cervical spine:** the patient was placed prone with Mayfield tongs for stabilization of the head. The head and neck were placed in flexion for easier decompression of the subaxial cervical spinal canal but returned to a neutral position before any fusion or instrumentation procedures. The arms and shoulders were placed at the patient´s side, with a gentle taping of the shoulders to the distal end of the bed to facilitate intraoperative radiographic visualization. The head of the operating table was elevated to 30 degrees of reverse trendelenburg positioning to reduce venous epidural bleeding. Knee flexion was done to prevent the patient from sliding inferiorly in this position. All bony prominences and peripheral nerves were carefully padded to prevent intraoperative neurapraxia.

A six-centimeter longitudinal midline incision was made, beginning at the C2 spinous process, and extending distally to the C7 spinous process. Midline dissection in the avascular median raphe was carried out using electrocautery. Subperiosteal dissection laterally from the spinous process was carried out to expose the lamina and lateral mass. The facet joint capsule was preserved. Ligamentum flavum was identified and detached from the lamina using a fine curet after confirmation of level by fluoroscopy. Laminectomy (and at least one-sided facetectomy) was performed to expose the dura and the lateral parts of the disc.

**Posterior approach to the dorsal spine:** the patient was put in the prone position on a Jackson table. A standard posterior midline approach with subperiosteal dissection of the paraspinal musculature was done over the involved levels, exposing the spine out to the transverse processes. Exposure was done at least one level above and below the fracture dislocated segment. Once the standard bony landmarks were identified, pedicle screws were inserted at the cephalad level. The number of screws used varied according to the severity of fracture dislocation and the number of involved segments. Precontoured rods were placed and fixed to the distal pedicle-screws. Before tightening the proximal screw nuts, distraction was applied using an instrumentation setting. Decompression of the spinal canal was performed in each patient because of the protruding fracture fragments and the accompanying neurological deficit. The ruptured disc and bone fragments in the spinal canal were removed through the posterolateral approach. The intervertebral disc and endplates were also removed. Once the bone graft bed was prepared, autologous bone harvest from the resected posterior arch was implanted and packed tightly into the gap. Posterolateral fusion was routinely performed before drainage and wound closure.

**Reporting:** results were reported according to The Strengthening the Reporting of Observational Studies in Epidemiology (STROBE) guidelines [13]. Results were evaluated using the ASIA grade for neurological outcome and functional outcome was assessed using the Barthel Scale/Index (BI), an ordinal scale used to measure performance in activities of daily living (ADL) measuring the degree of assistance required by an individual on 10 items of mobility and self-care.

The neurological outcome of this study was defined as “improved neurologic outcome” if there was at least a 1-grade AIS improvement at 6 months´ follow-up. Complete recovery was defined as an ASIA grade of E at 6 months´ follow-up. Incomplete recovery was defined as an ASIA grade of B, C or D at 6 months´ follow-up. No recovery was defined to have occurred if there was no improvement in ASIA grade from admission to 6 months´ follow-up. For assessing the functional status or degree of dependency at 6 months, Barthel index score of 0-20 was defined as total dependence, 21-60 as severe dependence, 61-90 as moderate dependence and 91-99 as slight dependence.

**Data analysis:** data were analysed using the Statistical Package for the Social Science (SPSS version 27.0; SPSS Inc., Chicago, IL, USA). Data were summarized as the mean and standard deviation for numerical variables and count and percentages for categorical variables. During analysis, P-values were computed for categorical variables using Chi-square and Fisher's exact test in accordance with the size of the dataset. P-value ≤ 0.05 was considered for statistical significance.

**Ethical approval:** the proposal for this study was approved by the Institutional Ethics Committee of our institution before the commencement of data collection. The reference number of approval is 4(6-11)-AGMC/Medical Education/Ethics Com./2018.

## Results

**Participants:** the participants included 30 patients with spinal cord injuries who were assessed for a period of 6 months following injury. All the patients underwent surgical stabilization of the involved spinal segments. Notably, patients with polytrauma and pre-existing neurological deficits were excluded from the study. The mean age of presentation was 44.27 years with a male predominance of 23 (76.7%). In our study, 18 (60%) patients suffered a fall from height while 10 (33.3%) patients were victims of road traffic accidents (Table 1).

**Table 1:** mode of injury and vertebral level of injury

Mode of injury	Number (n)	Percentage (%)
Fall from height	18	60.0
Road traffic accident	10	33.3
**Vertebral level of injury**	**Number (n)**	**Percentage (%)**
Cervical	14	46.7
Thoracolumbar	16	53.3

The mean transport time to the hospital was 3.27 days. Twelve (40%) patients reached our centre in less than 24 hours after injury, while 14 (46.7%) patients reached within a week and 4 (13.3%) patients reached more than a week post-injury. The thoracolumbar spinal cord was more commonly injured with 16 (53.3%) patients compared to cervical spinal cord injury patients at 14 (46.7%). Central cord syndrome was the most common single injury pattern, followed by burst fracture of first lumbar vertebra. Ten (33.3%) patients had ASIA grade A, 4 (13.3%) patients had ASIA grade B, 6 (20%) patients had ASIA grade C, and 10 (33.3%) patients had ASIA grade D at admission (Table 2).

**Table 2:** American Spinal Injury Association (ASIA) grade at admission and 6 months post-injury

ASIA grade	Number at admission	Number at discharge
A	10	8
B	4	2
C	6	3
D	10	7
E	-	8
Total	30	30

**Figure 1:**
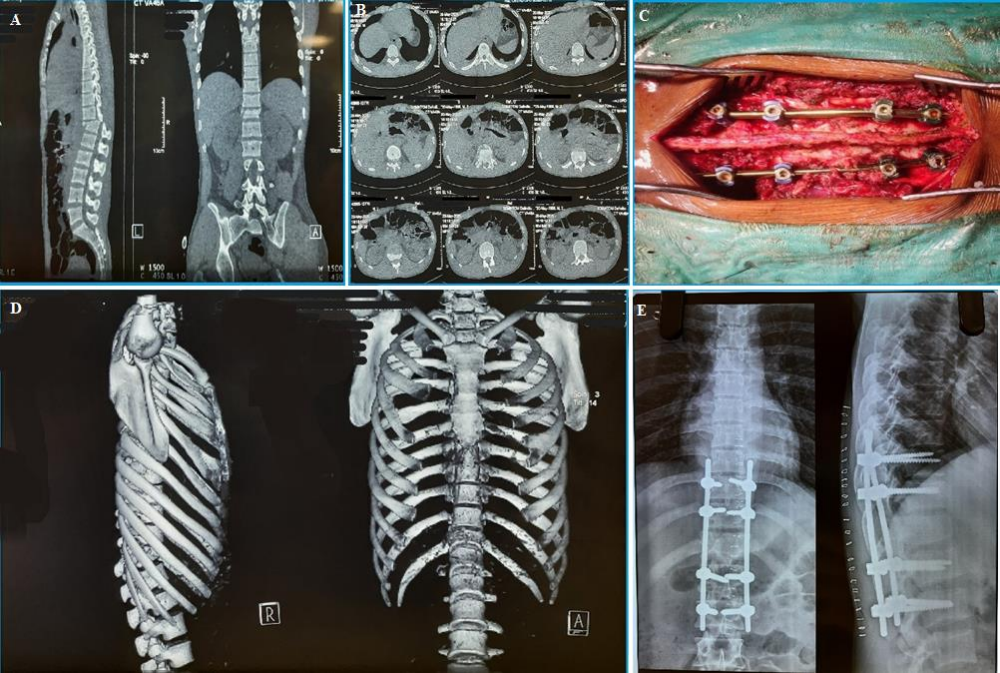
(A,B,C,D,E) degree of dependency according to Barthel score at 6 months

In our study, 13 (43.3%) patients underwent operative procedures within 1 week, 13 (43.3%) patients within 1-2 weeks, and 4 (13.3%) more than 2 weeks following admission. The mean time interval between admission and surgery was 8.73 days. 13 (43.3%) patients underwent operative procedures within 1 week, 13 (43.3%) patients within 1-2 weeks, and 4 (13.3%) more than 2 weeks following admission. Ten (33.3%) patients underwent surgery through the anterior approach and 20 (66.7%) patients through the posterior approach. A C5-C6 anterior cervical discectomy and fusion was the most performed procedure followed by T12-L2 posterior instrumented stabilization and decompression (Figure 1).

**Figure 2:**
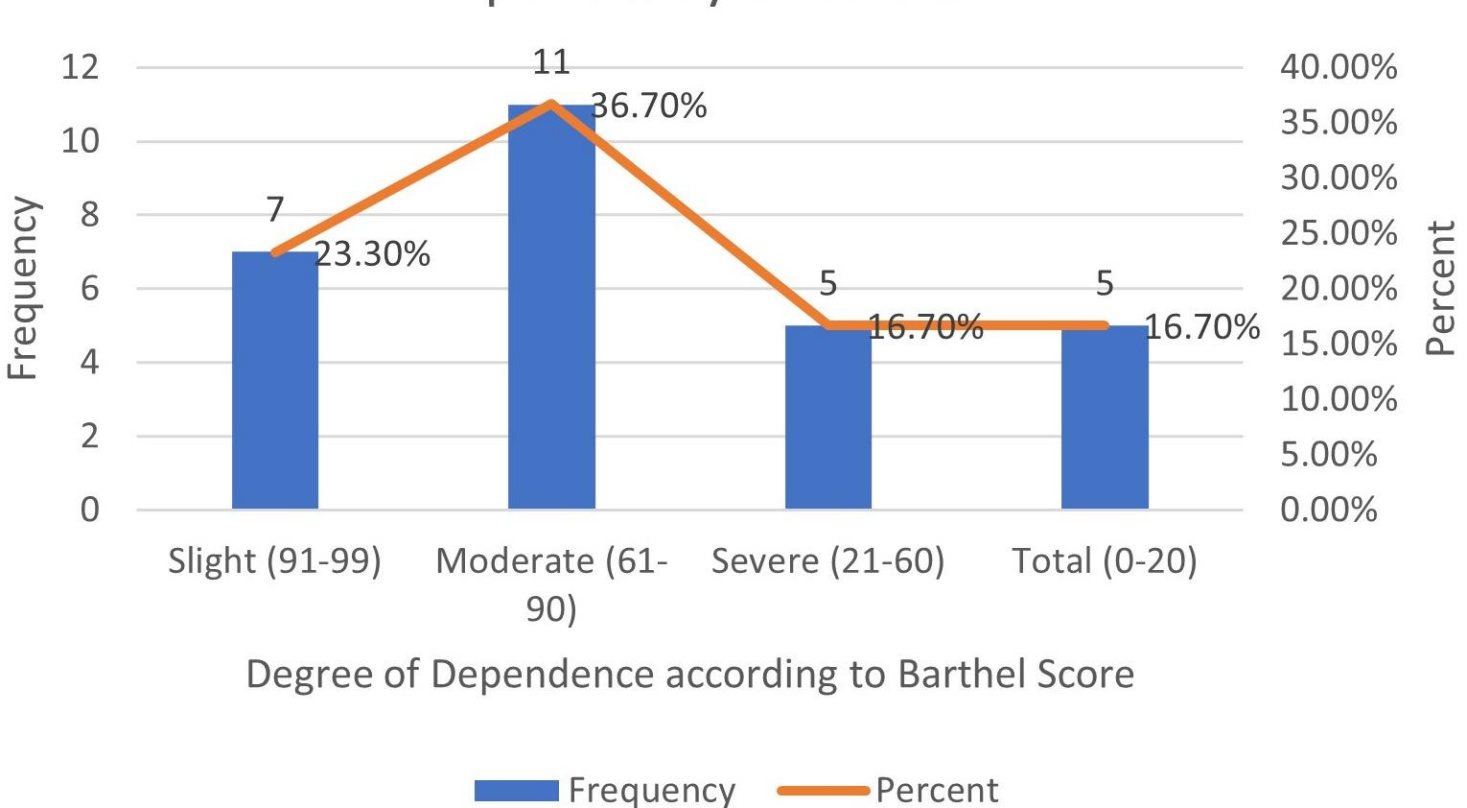
*Arbeitsgemeinschaft für Osteosynthesefragen* (AO) type C translation injury with American Spinal Injury Association (ASIA) A neurology

The mean duration of hospital stay was 23 days. Complications were observed in 33.3% of patients, like urinary tract infections, surgical site infections, dysphagia, and pressure sores. Eight (26.7%) patients had ASIA grade A, 2 (6.7%) patients had ASIA grade B, 3 (10%) patients had ASIA grade C, 7 (23.3%) patients had ASIA grade D and 8 (26.7%) patients had ASIA grade E at 6 months´ follow-up. The mean Barthel score observed was 65 with a median of 80, ranging from 20 (minimum) to 95 (maximum). In our study, 7 (23.3%) patients had slight dependence, 11 (36.7%) patients had moderate dependence, 5 (16.7%) patients had severe dependence, and 5 (16.7%) patients had total dependence at 6 months´ follow-up (Figure 2). Eight (26.7%) patients had complete recovery, seven (23.3%) patients had incomplete recovery and 15 (50.0%) patients had no recovery at the end of 6 months follow-up. A total of 18 (60%) patients had favourable functional outcome.

ASIA grade at admission was found to be significantly associated with the functional outcome at 6 months´ follow-up (P-value: <0.0001). Neurological outcome was found to be significantly associated with functional outcome at 6 months (P-value: <0.0001).

## Discussion

Developing countries face a unique set of challenges in managing patients with traumatic spinal cord injury. In contrast to countries with advanced trauma care, there is a lack of trained emergency response paramedical staff. This results in injudicious handling of patients, often converting an incomplete spinal cord injury into a complete one. Also, the medical care provided at most hospitals where these patients arrive at initially is suboptimal. In the present study, 30 cases of spinal cord injuries were operated and their neurological and functional outcomes were compared with other studies. The average follow-up duration was 6 months.

A cross-sectional study in Nepal conducted on 130 traumatic spinal cord injury patients calculated the prevalence of traumatic spinal injury to be 102 (78.46%) [14]. Spinal cord injury is common in young males in the age group of 31-40 years which reflects occupational factors and occur most commonly due to a fall from height. This contrasts with the epidemiological data from developed nations where road traffic accidents constitute the majority of the causal mechanism of injury. Preferential thoracolumbar and lumbar injuries in falls, lower survival rates of cervical injuries, and the relative inability of fresh cervical injuries to travel large distances may be some of the reasons for the higher proportion of thoracolumbar injuries in our study. Operative delay primarily due to economic constraints and delay in the provision of surgical theater reflect the major challenges in managing spinal cord injuries.

The total number of patients with complete injury was similar to the study by Agarwal *et al*. [15]. The almost non-existent pre-hospital care, inadequate emergency medical services in the facility providing initial treatment, the delay in reaching the facility providing definitive treatment may be responsible for the higher incidence of complete injuries observed in our study. In our study, the average duration of hospital stay was comparable to that observed by different studies. Prolonged hospital stay reflects the delay in provision of surgical theater, and mobilization of finance and resources required for surgery.

A retrospective study of dorsal and lumbar spine injury patients conducted in Kathmandu, Nepal on 91 patients found that twenty-seven patients had complete deficit, 32 incomplete deficit, and 32 normal neurology. Overall, 53.1% of neurologically incomplete deficit patients improved if operated within 30 days. No neurological improvement was seen in the 27 complete deficit patients [16]. In our study, the mean time interval between admission and surgery was 8.73 days which may not be acceptable in developed countries. However, despite the surgical delay, there was an improvement in functional outcome which is key to a successful rehabilitation.

Complications were observed in 10 (33.3%) patients in our study. Urinary tract infection, surgical site infection, and dysphagia were observed in 2 patients each, and pressure sores, and hoarseness of voice in 1 patient each. Two patients expired: 1 suffering from pneumonia and another from septicemia during the study period. Both cases had cervical spinal cord injury with complete neurological deficit. All complications were managed conservatively with good results. Chacko *et al*. [17] observed very high numbers of urinary tract infections (66.4%) and pressure sores (32.4%) and opined that adequate and effective care for spinal injured cannot be given in a general hospital ward and requires dedicated spinal injury units.

The mortality observed in our study was similar to other studies, considering the relative paucity of intensive care unit facilities for postoperative management. In our study, 8 (26.7%) patients had complete recovery, 7 (23.3%) patients had Incomplete recovery, and 15 (50.0%) patients had no recovery at 6 months follow-up. Agarwal *et al*. in their study reported complete recovery in 27.6% of patients, incomplete recovery in 31.9% of patients, and no recovery in 25.5%. Chacko *et al*. reported recovery in 31.2% of patients with neurological deficits. ASIA grade at admission was found to be significantly associated with the neurological outcome and functional outcome at 6 months follow-up.

There are significant challenges in managing spinal cord injuries in India which can be addressed by designing a quick referral system so that patients can be immediately shifted to higher centers with dedicated spine units. Provision of surgical theater, an inclusive spinal injury policy, public awareness programs on prevention of spine injuries, provision of trained paramedical staff to extricate patients from the site of injury, proper immobilization during transport, and lastly, legislation so that workers are provided adequate safety harnesses to minimize occupational injury can show the way forward to improving the lives of survivors of spinal cord injury.

**Limitations:** this was a single hospital-based analysis. There was no control group to compare outcomes. Also, pediatric patients and patients with concomitant life-threatening injuries were not included in the study. Long-term functional and radiological outcomes, rehabilitation, and societal and vocational integration of the patients were not included in the study.

## Conclusion

After surgery nearly half of the patients had an improvement in their neurology, and functional outcome was favorable which suggests that surgery still holds the key to a better functional and rehabilitation outcome. A quick referral system or provision of trained spine surgeons in the peripheral hospitals, early and frequent access to operating rooms, an inclusive spine injury policy, and public awareness programs on the prevention of spine injuries and proper immobilization during transport can show the way forward to improving the lives of survivors of spinal cord injury in our country.

### What is known about this topic

- *Medical and pharmacological management of spinal cord injuries is controversial;*
- *Surgical management or decompression in acute spinal cord injury is often the treatment of choice for neurological recovery*.

### What this study adds

- *Surgical management holds the key to better functional and rehabilitation ooutcomes in acute spinal cord injuries;*
- *Neurological status (ASIA grade) at admission was found to be significantly associated with the neurological outcome and functional outcome at 6 months follow-up in acute spinal cord injuries*.
